# Alzheimer's disease susceptibility genes *APOE* and *TOMM40*, and brain white matter integrity in the Lothian Birth Cohort 1936^☆^

**DOI:** 10.1016/j.neurobiolaging.2014.01.006

**Published:** 2014-06

**Authors:** Donald M. Lyall, Sarah E. Harris, Mark E. Bastin, Susana Muñoz Maniega, Catherine Murray, Michael W. Lutz, Ann M. Saunders, Allen D. Roses, Maria del C. Valdés Hernández, Natalie A. Royle, John M. Starr, David. J. Porteous, Joanna M. Wardlaw, Ian J. Deary

**Affiliations:** a Centre for Cognitive Ageing and Cognitive Epidemiology, University of Edinburgh, Edinburgh, UK; b Brain Research Imaging Centre, Division of Neuroimaging Sciences, University of Edinburgh, Edinburgh, UK; c Department of Psychology, University of Edinburgh, Edinburgh, UK; d Medical Genetics Section, University of Edinburgh Centre for Genomics and Experimental Medicine, Western General Hospital, Edinburgh, UK; e MRC Institute of Genetics and Molecular Medicine, Western General Hospital, Edinburgh, UK; f Scottish Imaging Network, A Platform for Scientific Excellence (SINAPSE) Collaboration, Department of Neuroimaging Sciences, The University of Edinburgh, Edinburgh, UK; g Department of Neurology, Joseph & Kathleen Bryan Alzheimer's Disease Research Center, Durham, NC, USA; h Duke University Medical Center, Durham, NC, USA; i Zinfandel Pharmaceuticals, Inc, Durham, NC, USA; j Alzheimer Scotland Dementia Research Centre, University of Edinburgh, Edinburgh, UK

**Keywords:** White matter, Cognitive ageing, Diffusion MRI, Tractography, *APOE*, *TOMM40*, Alzheimer's disease

## Abstract

Apolipoprotein E (*APOE*) ε genotype has previously been significantly associated with cognitive, brain imaging, and Alzheimer's disease-related phenotypes (e.g., age of onset). In the *TOMM40* gene, the rs10524523 (“523”) variable length poly-T repeat polymorphism has more recently been associated with similar ph/enotypes, although the allelic directions of these associations have varied between initial reports. Using diffusion magnetic resonance imaging tractography, the present study aimed to investigate whether there are independent effects of apolipoprotein E (*APOE*) and *TOMM40* genotypes on human brain white matter integrity in a community-dwelling sample of older adults, the Lothian Birth Cohort 1936 (mean age = 72.70 years, standard deviation = 0.74, N approximately = 640–650; for most analyses). Some nominally significant effects were observed (i.e., covariate-adjusted differences between genotype groups at *p* < 0.05). For *APOE*, deleterious effects of ε4 “risk” allele presence (vs. absence) were found in the right ventral cingulum and left inferior longitudinal fasciculus. To test for biologically independent effects of the *TOMM40* 523 repeat, participants were stratified into *APOE* genotype subgroups, so that any significant effects could not be attributed to *APOE* variation. In participants with the *APOE* ε3/ε4 genotype, effects of *TOMM40* 523 status were found in the left uncinate fasciculus, left rostral cingulum, left ventral cingulum, and a general factor of white matter integrity. In all 4 of these tractography measures, carriers of the *TOMM40* 523 “short” allele showed lower white matter integrity when compared with carriers of the “long” and “very-long” alleles. Most of these effects survived correction for childhood intelligence test scores and vascular disease history, though only the effect of *TOMM40* 523 on the left ventral cingulum integrity survived correction for false discovery rate. The effects of *APOE* in this older population are more specific and restricted compared with those reported in previous studies, and the effects of *TOMM40* on white matter integrity appear to be novel, although replication is required in large independent samples.

## 1 Introduction

The apolipoprotein E (*APOE*) gene is located on chromosome 19q13.2 and is 3.7 kilobases (KB) long. *APOE* ε genotype is composed of 2 single-nucleotide polymorphisms (SNPs): rs429358, which causes a Cys130Arg substitution; and rs7412, which causes an Arg176Cys substitution; different combinations of the rs429358 and/or rs7412 SNPs form the ε2 (Cys/Cys, respectively), ε3 (Cys/Arg), and ε4 (Arg/Arg) genotypes (NCBI website, 2012a; Ringman and Cummings, 2009). Of these, the ε3 allele is the most common (frequency ∼78.3% in Caucasians), followed by ε4 (∼14.5%) and ε2 (∼6.4%), although these frequencies vary between populations (Eisenberg et al., 2010).

*APOE* plays a role in the transport and metabolism of lipids in the human body and brain (Bu et al., 2009; Corder et al., 1994). The ε4 allele of *APOE* is the “risk” variant for several phenotypes compared with ε3 (“neutral”), and ε2 (generally considered “protective”, although less consistently). These phenotypes include risk of Alzheimer's disease (AD) (Corder et al., 1994), less successful cognitive aging (Deary et al., 2004; Wisdom et al., 2011), differences in brain structure (e.g., atrophy; Biffi et al., 2010), and functional connectivity (Trachtenberg et al., 2012); vascular pathologies such as hyperlipidemia, coronary heart disease and stroke (Lahoz et al., 2001), and brain microbleeds (Schilling et al., 2013). It is not clear to what extent associations between *APOE* variants and worse cognitive aging in cross-sectional and longitudinal studies reflect preclinical “prodromal” AD (Bretsky et al., 2003; Deary et al., 2004).

There might be complexities in how the *APOE* ε4 allele is associated with clinical onset of AD or cognitive decline (Johnson et al., 2011). Other genetic variants aside from *APOE*, possibly in linkage disequilibrium with it, may play a role. The translocase of the outer membrane of 40 (*TOMM40*) gene is located adjacent to *APOE* and covers 12.5 KB on chromosome 19q13 (NCBI website, 2012b). Several SNPs in the *APOE* and *TOMM40* genes are in strong linkage disequilibrium; for example, rs429358 and 36 SNPs within ±1.17 KB of the *APOE* region including 15 *TOMM40* SNPs; average D′ = 0.91, r^2^ = 0.22, n = 1262 (Yu et al., 2007). The *TOMM40* locus encodes for a channel-forming subunit of the translocase of the outer mitochondrial membrane complex (Humphries et al., 2005). This complex imports precursor proteins into mitochondria (Koehler et al., 1999). Mitochondrial dysfunction may play a significant role in cognitive decline and AD-related pathology (“The mitochondrial cascade hypothesis”; Swerdlow and Kahn, 2004). *APOE* and *TOMM40* may interact to affect aspects of mitochondrial function although mechanistically it is unclear exactly how (Roses et al., 2010).

The rs10524523 locus (hereafter “523”) in the *TOMM40* gene is characterized by a variable number of T residues (“poly-T repeats”) that can be classified into 3 different lengths: “short” (<20; “S”), “long” (20–29; “L”), and “very-long” (≥30; “VL”; Lutz et al., 2010). Roses et al. (2010) showed with phylogenetic mapping analyses that *TOMM40* 523 poly-T repeat length was strongly linked to *APOE* ε genotype in humans: ε4 is most commonly linked to the 523 L allele, with ε3 linked to either S or VL alleles in different evolutionary clades. The rarer ε2 allele appeared similar to ε3 although further research is required in large samples (Roses et al., 2010).

Studies have tested for association between *TOMM40* 523 repeat length and different brain-related phenotypes independently from *APOE*. For example, age of AD onset (Roses et al., 2010) and worse cognitive aging (Schiepers et al., 2012), were examined. However, reports vary in showing protective (Johnson et al., 2011), null (Chu et al., 2011), or deleterious (Cruchaga et al., 2011) effects of the S allele. See Roses et al. (2013) for a discussion of early studies.

Diffusion-tensor magnetic resonance imaging (MRI) and quantitative tractography allow examination of brain white matter microstructure in vivo in specific white matter tracts thought to relate to cognitive functions (Behrens et al., 2007; Pierpaoli et al., 1996). Diffusion-tensor imaging (DTI) measures the magnitude and directional coherence of water molecule diffusion and, because water molecule diffusion is preferentially constrained along the principal fiber direction by axonal membranes and myelin sheaths, this property can be used to assess white matter structural integrity (Behrens et al., 2007; Pierpaoli et al., 1996). Fractional anisotropy (FA) is an example of a common DTI-derived metric, and reflects the level of directional coherence of water molecule diffusion (Pierpaoli et al., 1996). Specifically, FA measures are high in healthy, structurally intact, coherently organized white matter, but fall in diseased tissue. Associations between the *APOE* gene and white matter integrity have been investigated previously (see Gold et al., 2012 for a review of significant findings; also Felsky and Voineskos, 2013). To our knowledge, the largest previous study had 203 participants (Westlye et al., 2012; mean age = 47.6 years, standard deviation = 14.9). That report found widespread differences in microstructural integrity depending on *APOE* status. Controlling for age and gender, ε3/ε4 carriers had lower white matter integrity (vs. ε3/ε3) in the brainstem, basal temporal lobe, internal capsule, anterior parts of the corpus callosum, forceps minor, superior longitudinal fasciculus, occipital, and corticospinal motor pathways (Cohen's *d* range = 0.77–0.79; “medium-large effects”). We found no studies that examined the independent effects of the *TOMM40* 523 poly-T repeat.

The present study aims to investigate the effects of *APOE* and *TOMM40* genotypes on brain white matter integrity as assessed using quantitative tractography in a large, age-homogenous sample of relatively healthy older people. Fourteen major projection, commissural, and association fiber tracts were examined that have previously been significantly associated with cognitive abilities in this sample (Penke et al., 2012).

## 2 Methods

### 2.1 Sample and procedure

The LBC1936 is a cohort of 1091 generally healthy community-dwelling adults, 1028 of whom completed the Moray House Test no.12 (MHT) of verbal reasoning as part of the Scottish Mental Survey 1947 at a mean age of 11 years. The recruitment and testing of this sample has been detailed in previous protocol papers (Deary et al., 2007, 2012). All the LBC1936 participants were born in 1936 and most resided in the Edinburgh (Lothian) area of Scotland when recruited in older adulthood. In the first wave of the LBC1936 study (“wave 1”), at around the age 70 years, they were retested on the MHT in addition to other detailed cognitive, sociodemographic, and physical assessments (Deary et al., 2007). Around 3 years later, 866 members of the cohort returned for re-testing in the second wave of the study (“wave 2”). At this point, in addition to repeating the wave 1 assessments, most of the participants also underwent detailed structural brain MRI (Wardlaw et al., 2011). At both waves, participants were screened for cognitive impairment with the Mini-Mental State Examination (MMSE), with scores under 24 used to indicate possible dementia (Folstein et al., 1975). Medical history was elicited via interview, including hypertension, diabetes, stroke, hypercholesterolemia, and history of any other vascular disease (e.g., heart attack). All subjects gave written, informed consent after the nature of the procedures had been explained to them.

### 2.2 Cognitive assessment

*Moray House Test*: This was completed in 1947 at a mean age of 11 years. This test of general cognitive ability has a 45-minute time limit and has a maximum score of 76, with a predominance of verbal reasoning items, and some numerical and visuospatial items also included (Deary et al., 2007). MHT scores were adjusted for age in days at the time of assessment, and standardized to an IQ score with a mean of 100 and a standard deviation of 15 for the whole LBC1936 sample.

### 2.3 Genotyping

For *APOE*, DNA was isolated from whole blood, and the target sequences for rs7412 and rs429358 were genotyped with TaqMan technology (Invitrogen website, 2012). These 2 SNPs form the *APOE* ε2/ε3/ε4 haplotype (commonly and herein simply “genotype”).

*TOMM40* 523 poly-T repeat length was genotyped by the laboratory of Dr. Ornit Chiba-Falek (Duke University, NC, USA) using a method described previously (Linnertz et al., 2012). Briefly, each genomic DNA sample was amplified by the polymerase chain reaction using fluorescently labeled forward 5′FAM-TGCTGACCTCAAGCTGTCCTG-3′ and reverse 5′-GAGGCTGAGAAGGGAGGATT-3′primers. Genotypes were determined on an ABI 3730 DNA Analyzer, using GeneMapper version 4.0 software (Applied Biosystems, Foster City, California, USA) for fragment analysis by the amplified fragment length polymorphism method validated for research studies and commercially available.

### 2.4 Diffusion MRI and tractography analysis

Participants underwent whole brain diffusion MRI acquired using a GE Signa Horizon HDxt 1.5-T clinical scanner (General Electric, Milwaukee, USA) equipped with a self-shielding gradient set (33 mT m^−1^ maximum gradient strength) and manufacturer-supplied 8-channel phased-array head coil (Wardlaw et al., 2011). The protocol consisted of 7 T2-weighted (b = 0 s mm^−2^) and sets of diffusion-weighted (b = 1000 s mm^−2^) axial single-shot spin-echo echo-planar volumes acquired with diffusion gradients applied in 64 noncollinear directions. Seventy-two contiguous slices of 2 mm thickness were collected with a field of view of 256 × 256 mm and acquisition matrix of 128 × 128, giving 2 mm isotropic voxel resolution.

The diffusion MRI data were preprocessed using FMRIB Sofware Library (FSL) tools (Oxford, UK; Nuffield Department of Clinical Neurosciences, 2012) to extract the brain, remove bulk patient motion and eddy current induced artifacts, and generate parametric maps of FA. Underlying tractography connectivity data were generated using BedpostX/ProbTrackX with the default settings of a 2-fiber model per voxel, and 5000 probabilistic streamlines with a fixed separation of 0.5 mm between successive points (Behrens et al., 2007).

Fourteen tracts were identified using probabilistic neighborhood tractography, an approach for automatic and reproducible tract segmentation (Clayden et al., 2007), as implemented in the TractoR package for fiber tracking analysis (Clayden et al., 2011; Tractography with R website, 2012). This method segments the same fasciculus-of-interest in different individuals by identifying the best-matched tract from a group of “candidate” tracts that most closely resembles a predefined reference tract in terms of both length and shape. This approach has advantages over tract-based spatial statistics in that each tract is segmented in native rather than standard space, therefore providing a better representation of specific tract anatomy in each individual (Bastin et al., 2013). Tracts assessed were the genu and splenium of corpus callosum, and bilateral anterior thalamic radiations, ventral and rostral cingulum bundles, and arcuate, uncinate, and inferior longitudinal fasciculi. Tract masks generated by this method were overlaid on the FA parametric map and tract-averaged values, weighted by the connection probability, were determined for each tract in every subject.

To ensure that the segmented tracts were anatomically plausible representations of the fasciculi-of-interest, a researcher visually inspected all tract masks blind to the other study variables and excluded those with aberrant or truncated pathways. In general, probabilistic neighborhood tractography was able to segment the 14 tracts-of-interest reliably in most of the subjects, with tracts that did not meet quality criteria, such as truncation or failing to follow the expected path, ranging from 0.3% for the splenium of the corpus callosum to 16% for the left anterior thalamic radiation, with a mean of 5%.

To permit principal components analysis (PCA) on the tractography data, participants with up to 2 missing values from specific tracts had data replaced with the mean value for that tract. PCA was conducted on tract-averaged water diffusion parameters for 12 pathways, giving a clear single-factor model for FA (*g*_*fa*_) that accounted for 38.78% of the overall variance. The ventral cingulum was not included in the PCA because the rostral and ventral cingula are subdivisions of the same tract. General white matter integrity factors have previously been found to be associated with cognitive abilities in this sample (Penke et al., 2010, 2012).

## 3 Statistical analysis

### 3.1 Statistical models

Three statistical models were used to investigate the effect of *APOE* and *TOMM40* genetic variants (the independent variables) on tract-averaged FA values (the dependent variables) for the 14 tracts and *g*_*fa*_. First, all models controlled for covariates of gender and age in days at neuroimaging (“model 1”). Significant effects were re-tested controlling for the additional covariate of age-11 IQ (“model 2”). This reduces the chance of results being affected by reverse causality, where differences in early-life cognitive ability might, perhaps via influence on health-related variables, influence brain changes in later life (Deary et al., 2007; Shenkin et al., 2003). Finally, significant effects were re-tested controlling for the following covariates in addition to those in model 2; self-reported history of hypertension, stroke, type-2 diabetes, hypercholesterolemia, and all-inclusive vascular disease (“model 3”; Schiepers et al., 2012). This reduces the chance that any significant associations occur as secondary to genetic associations with vascular pathology.

An online calculator was used to perform tests of Hardy–Weinberg equilibrium and determine minor allele frequencies (Calculator; Hardy Weinberg equilibrium, 2012). Data were otherwise analyzed with IBM Statistical Package for the Social Sciences, version 19.0 (SPSS Inc, Chicago, USA). Specifically, univariate general linear models (GLM) tested the fixed effects of separate *APOE* and *TOMM40* genotypes upon the outcome variables, namely white matter integrity parameters. Outliers of more than 3 standard deviations from mean values were removed from age-11 IQ (n = 7). To protect against type 1 errors, false discovery rate (FDR) was used to estimate the number of significant findings in the context of multiple testing (Benjamini et al., 1995). A Microsoft excel program (Pike, 2011) was used to conduct classical 1-stage FDR based on associations with white matter integrity. All *p-*values are raw unless stated as being FDR-adjusted. *p*-values < 0.05 were considered to be “nominally” significant. Final results did not differ whether FDR was applied separately to individual sets of analyses (i.e., A*POE* and then *TOMM40* sub-analyses), or to a collated list of all analyses.

### 3.2 APOE analysis

The *APOE* ε genotype is composed of any 2 of the ε2 (protective), ε3 (neutral), and ε4 (risk) alleles. The first analytic step tested the effects of *APOE* ε4 allele presence versus absence, that is, pooled ε2/ε4, ε3/ε4, and ε4/ε4 genotypes versus pooled ε2/ε2, ε2/ε3, and ε3/ε3 (“step 1”). The second analytic step tested genotypes, which may be protective for neurodegenerative pathology, compared with the neutral genotype; that is, pooled ε2/ε3 and ε2/ε2 versus ε3/ε3 (“step 2”; Deary et al., 2004; Luciano et al., 2009).

### 3.3 TOMM40 analysis

The variable-length poly-T repeat rs10524523 (“523”) was split into 3 categories: “S” (<20 T residues), “L” (≥20), and “VL” (≥30) (Lutz et al., 2010), of which the S allele may or may not be protective in terms of neurodegenerative pathology (Bruno et al., 2011; Chu et al., 2011). In the first analytic step, in the whole sample, a GLM tested for a significant effect of *TOMM40* 523-genotype (i.e., S/S; S/L; L/L; L/VL; VL/VL; “step 1”).

To investigate the effects of *TOMM40* 523 repeat length independent of biological variation in *APOE* genotype, analysis then focused separately on 2 different large *APOE* ε genotype subgroups; firstly participants with the ε3/ε4 genotype (“step 2”). Devi et al. (2006) reported that this genotype had the highest accumulation of amyloid precursor protein in AD brain mitochondria (vs. ε3/ε3 and ε4/ε4 genotypes; assessed by immunoblot analysis), and this correlated strongly with 2 indicators of mitochondrial dysfunction (cytochrome C oxidase activity and reactive oxygen species hydrogen peroxide; “H_2_O_2_”). In terms of a possible interaction between amyloid precursor protein accumulation and mitochondria translocase processes, it is not known if the S allele may offset or interact biologically with the ε3/ε4 genotype (Bruno et al., 2011). Finally, analysis focused on participants with the neutral *APOE* genotype (ε3/ε3) (“step 3”), because this eliminates variance associated with protective and risk *APOE* alleles (Roses et al., 2010). Examining the effects of *TOMM40* 523 in *APOE* subgroups means that any significant associations cannot logically be attributed to *APOE* genotypic variation.

In large samples of Caucasians, linkage between the *APOE* ε genotype and *TOMM40* 523 length (i.e., ε4 links primarily to “L”, ε3 primarily to “S” or “VL”) is such that in the *APOE* ε3/ε3 genotype, relatively few L carriers would be predicted while in the ε3/ε4 genotype typically 1 L allele would be predicted in addition to either an S or VL allele (Linnertz et al., 2012). Slight errors in poly-T repeat length measurement may occur through polymerase chain reaction “slippage” and this may result in repeat lengths that are close to the L and/or VL boundary being incorrectly classified (Linnertz et al., 2012). To attempt to control for this, in steps 2 and 3, the L and VL alleles were pooled into an “L*” group; participants with the S/S genotype were compared with those carrying only 1 S allele (pooled S/L and S/VL; hereinafter S/L*), and also against participants carrying no S alleles (pooled L/L, L/VL, and VL/VL; hereinafter L*/L*; Caselli et al., 2012). A GLM therefore tested for effects of S-allele group (S/S; S/L*; L*/L*) on brain white matter integrity variables in steps 2 and 3 (*APOE* ε3/ε4 and ε3/ε3 subgroups, respectively).

## 4 Results

### 4.1 Descriptive statistics

Of the 1091 total LBC1936 participants, 866 attended waves 1 and 2, and 731 underwent neuroimaging. Individuals who reported being ambidextrous or left handed at either “wave 1” or “wave 2” (n = 50), or had MMSE scores below 24 (n = 5), or did not complete the MMSE at wave 2 (n = 1) were excluded. No participants had a history of dementia. Overall, this left 675 participants, of which 642 and 652 participants had successful genotyping for *APOE* and *TOMM40*, respectively.

*APOE* had allele frequencies of ε2 = 7.4%, ε3 = 77.0%, and ε4 = 15.6%, with genotype frequencies of: ε2/ε2 = 2 (0.3%), ε2/ε3 = 77 (12.0%), ε2/ε4 = 14 (2.2%), ε3/ε3 = 376 (58.6%), ε3/ε4 = 160 (24.9%), and ε4/ε4 = 13 (2.0%). *TOMM40* 523 had allele frequencies of S = 41.3%, L = 15.3%, and VL = 43.4%, with genotype frequencies of S/S = 102 (15.6%), S/L = 94 (14.4%), S/VL = 240 (36.8%), L/L = 15 (2.3%), L/VL = 76 (11.7%), and VL/VL = 125 (19.2%). Exact tests confirmed that *APOE* and *TOMM40* were in Hardy–Weinberg equilibrium (*p*-values = 0.656 and 0.273, respectively). Sample demographics for different *APOE* and *TOMM40* genotypes are displayed in Supplementary Table 1.

### 4.2 APOE and quantitative tractography

For the *APOE* ε4 present versus absent comparison, significant effects were found for 2 tracts, where presence of the ε4 allele was associated with poorer white matter integrity in the predicted direction (see Tables 1 and 3).

The first significant effect was found in the right ventral cingulum (F [1, 570] = 5.48, *p* = 0.020, and partial η^2^ = 0.010). This survived correction for age-11 IQ (F [1, 535] = 4.17, *p* = 0.042, and partial η^2^ = 0.008), and vascular disease history (F [1, 530] = 3.90, *p* = 0.049, and partial η^2^ = 0.007).

The second significant effect was found in the left inferior longitudinal fasciculus (F [1, 575] = 5.04, *p* = 0.025, and partial η^2^ = 0.009). This survived correction for age-11 IQ (F [1, 539] = 7.30, *p* = 0.007, and partial η^2^ = 0.013), and vascular disease history (F [1, 534] = 7.00, *p* = 0.008, and partial η^2^ = 0.013).

There was no main effect of the pooled ε2/ε3 and ε2/ε2 genotypes (vs. ε3/ε3) on the white matter integrity of any of the tracts analyzed (see Table 1).

### 4.3 TOMM40 523 length and quantitative tractography

In the whole sample (step 1), 2 significant effects of *TOMM40* 523 genotype were found (see Tables 2 and 3). The first significant effect was found in the left ventral cingulum (F [5, 564] = 2.60, *p* = 0.025, and partial η^2^ = 0.022). This effect survived correction for additional covariates of age-11 IQ (F [5, 531] = 2.57, *p* = 0.026, and partial η^2^ = 0.024), and vascular disease history (F [5, 526] = 2.79, *p* = 0.017, and partial η^2^ = 0.026). Post hoc tests showed that the S/L genotype had significantly lower FA compared with S/S (*p* = 0.029), S/VL (*p* = 0.008), L/L (*p* = 0.033), and L/VL (*p* = 0.003) genotypes (all other comparisons were *p* > 0.05).

The second significant effect was found in the right rostral cingulum (F [5, 568] = 2.99, *p* = 0.011, and partial η^2^ = 0.026). This effect survived correction for age-11 IQ (F [5, 533] = 3.73, *p* = 0.003, and partial η^2^ = 0.034), and vascular disease history (F [5, 528] = 3.62, *p* = 0.003, and partial η^2^ = 0.033). Post hoc tests showed that the S/L genotype had significantly lower FA compared with the S/VL (*p* = 0.011), and L/VL (*p* = 0.026) genotypes, with VL/VL having significantly lower FA compared with L/VL (*p* = 0.017; all other comparisons were *p* > 0.05).

To determine whether association between *TOMM40* 523 length and white matter tract integrity was driven by linkage with *APOE*, the previously mentioned associations were re-tested controlling for presence of the ε4 allele. Controlling for age, gender, and *APOE* ε4 presence, the main effect of *TOMM40* 523 length remained for both right rostral cingulum FA (F [5, 547] = 3.00, *p* = 0.011, and partial η^2^ = 0.024) and left ventral cingulum FA (F [5, 544] = 3.45, *p* = 0.004, and partial η^2^ = 0.031).

In ε3/ε4 carriers (step 2), 4 significant effects of *TOMM40* 523 length were found; these suggested a deleterious effect of the S/L* genotype versus L*/L* (see Table 2). This indicates that possessing an S allele is associated with lower white matter integrity compared with possessing only L or VL alleles (as pooled into the “L*” group). Note that the ε3/ε4 group had no S/S homozygotes.

The first significant effect was found for *g*_*fa*_ (F [1, 122] = 4.95, *p* = 0.028, and partial η^2^ = 0.039). This survived correction for additional covariates of age-11 IQ (F [1, 114] = 5.35, *p* = 0.023, and partial η^2^ = 0.045) and vascular disease history (F [1, 109] = 5.84, *p* = 0.017, and partial η^2^ = 0.051).

The second significant effect was found in the left uncinate fasciculus (F [1, 117] = 4.16, *p* = 0.044, and partial η^2^ = 0.034), which did not survive the addition of the covariate of age-11 IQ (F [1, 108] = 3.68, *p* = 0.058, and partial η^2^ = 0.033). However, the initial significant effect remained when including vascular disease history but not age-11 IQ as a covariate, (F [1,112] = 4.12, *p* = 0.045, and partial η^2^ = 0.035).

The third significant effect was found in the left rostral cingulum (F [1, 133] = 5.51, *p* = 0.020, and partial η^2^ = 0.040). This survived correction for additional covariates of age-11 IQ (F [1, 124] = 5.50, *p* = 0.021, and partial η^2^ = 0.042) and vascular disease history (F [1, 119] = 5.83, *p* = 0.017, and partial η^2^ = 0.047).

The fourth and final significant effect was found in the left ventral cingulum (F [1, 136] = 13.35, *p* < 0.001, and partial η^2^ = 0.089) and remained after correction for additional covariates of age-11 IQ (F [1, 125] = 14.40, *p* = <0.001, and partial η^2^ = 0.103) and vascular disease history (F [1, 120] = 15.75, *p* = <0.001, and partial η^2^ = 0.116).

No significant effects of the *TOMM40* 523 poly-T repeat length polymorphism were found at step 3 (i.e., in the ε3/ε3 genotype) (Table 2).

### 4.4 Correction for multiple testing

With FDR correction, all nominally significant (uncorrected) effects attenuated to nonsignificance except for the main effect of *TOMM40* 523 S/L* versus L*/L* on left ventral cingulum FA in *APOE* ε3/ε4 carriers (models 1, 2, and 3; FDR-adjusted *p*-values all = 0.017).

## 5 Discussion

### 5.1 Overview

The present study investigated the effects of variants in 2 genes upon brain white matter tract integrity in a large sample of nondemented, community-dwelling people in their early 70s. These gene loci were *APOE* ε and the *TOMM40* 523 poly-T repeat. The current report is the largest examination of the *APOE* locus and white matter integrity in a single study (Gold et al., 2012; Westlye et al., 2012) and therefore adds a significant amount of new data to the literature. No previous studies have examined *TOMM40* 523 in relation to this phenotype.

The present study found significant effects of the *APOE* ε4 risk allele in the predicted deleterious direction on: (1) the right ventral cingulum; and (2) the left inferior longitudinal fasciculus. The ventral cingulum is a parieto-occipital tract connecting the cingulate cortex with parahippocampal gyri and terminating in the anterior part of the medial temporal lobes. The left inferior longitudinal fasciculus connects occipital and temporal areas including the hippocampus (Catani and Thiebaut de Schotten, 2008).

*TOMM40* 523 poly-T repeat length genotype had significant effects in the whole sample and in the subgroup of participants that possessed the *APOE* ε3/ε4 genotype. In the whole sample, significant effects were found in the left ventral cingulum and the right rostral cingulum, both primarily driven by the S and/or L genotype being associated with significantly lower FA compared with other *TOMM40* 523 length genotypes. These significant associations survived statistical correction for presence of the *APOE* ε4 allele, however were not significant in the subgroup of participants with the “neutral” ε3/ε3 genotype, suggesting they are either: (1) unlikely to be truly independent of *APOE* genotype, and reflect type 1 error; or (2) sensitive to reductions in sample size. Larger samples of individuals with the ε3/ε3 genotype would be required to address this further.

In *APOE* ε3/ε4 carriers, a significant deleterious effect of possessing an S allele (vs. not) was found in: the (1) left uncinate fasciculus; (2) left rostral cingulum; (3) left ventral cingulum; and (4) general factor of white matter integrity (*g*_*fa*_). The uncinate fasciculus connects the anterior temporal lobe to medial and lateral orbitofrontal cortex, whereas the rostral cingulum projects from the cingulate to orbitofrontal cortices (Catani and Thiebaut de Schotten, 2008). The *g*_*fa*_ parameter, which is determined from PCA of the tract-averaged FA values for 12 major fiber pathways in each subject, shows that the integrity of white matter is to a substantial degree shared across tracts throughout the brain, possibly indicating shared influences (Lopez et al., 2012; Penke et al., 2012).

Most of the reported associations remained when corrected for history of vascular disease (e.g., history of stroke, etc.) and childhood intelligence. Corrected for multiple testing, only the deleterious effect of the *TOMM40* 523 S allele (vs. pooled noncarriers, in the *APOE* ε3/ε4 subgroup analysis) upon left ventral cingulum integrity remained significant. White matter diffusion tensor phenotypes are highly correlated as indicated by the *g*_*fa*_ factor. This can make correction for multiple testing overly conservative; therefore, for different reasons, nominal and adjusted effects require caution in their interpretation (Nyholt et al., 2001; Williams and Haines, 2011). All findings reported therefore require replication in large independent samples.

### 5.2 *APOE* ε genotype

The present study partially replicates previous associations between ε4 and tracts associated with temporal lobe structures (before correction for multiple testing); specifically the inferior longitudinal fasciculus (Gold et al., 2010; Smith et al., 2010), and ventral cingulum (Smith et al., 2010; “posterior cingulum”). It is possible that the effects of *APOE* seen here are indicative of prodromal AD in the current sample. However, it is unclear why the left inferior longitudinal fasciculus and right ventral cingulum would be particularly vulnerable to the effects of the *APOE* ε4 allele, and not the integrity of other white matter tracts; these findings may reflect a degree of type-1 error as reflected by their attenuation to nonsignificance when corrected with FDR. Common genetic variants typically have small effect sizes, and it is possible that the current sample–while relatively large–may not be sufficiently powered to detect small effects.

The present study is the largest assessment of *APOE* and white matter integrity to date and reports more circumscribed effects of ε4 allele possession compared with previous reports (Gold et al., 2012). This could relate to the relatively large sample size and homogenous age ranges assessed here compared with previous studies. Larger samples are likely to be more reliable, and wide age ranges could include the cumulative influence of subtle age-related processes; correlation between age and brain phenotypes is unlikely to be completely unique and rather may be via processes *associated* with chronological age; it is therefore unlikely that statistically controlling for age would completely capture this (Hofer and Sliwinski, 2001). Discrepancies may also relate to differences between image analysis methods (e.g., tract-based spatial statistics; Westlye et al., 2012) versus probabilistic neighborhood tractography used here. The present study does not interrogate white matter integrity across the whole brain and therefore cannot exclude the possibility of significant ε4 effects in regions that were not assessed, such as the fornix. Regardless, the nominally significant effects observed here were weaker and more specific than would have been expected when compared with the “medium-large” effects reported previously (Gold et al., 2012; Westlye et al., 2012).

### 5.3 TOMM40 523 poly-T repeat

Associations between the *TOMM40* 523 poly-T repeat and white matter integrity have not been investigated by previous studies. The previously mentioned results indicate an independent deleterious effect of the *TOMM40* 523 S allele in *APOE* ε3/ε4 but not ε3/ε3 genotypes. This association is independent in the sense that any deleterious effects of *TOMM40* 523 S allele possession (vs. non-possession) cannot be causally attributed to linkage with *APOE* ε alleles, because a specific stable subgroup was analyzed. Similar to *APOE*, the tracts that showed negative association with the S allele have been implicated in early AD pathology; the (left) uncinate fasciculus, (left ventral) cingulum, and (left rostral) cingulum (Gold et al., 2010; Heise et al., 2011; Smith et al., 2010). As with *APOE*, it is unclear why these specific tracts (and not others) would be exclusively affected by *TOMM40* 523 length. It is possible that the *TOMM40* 523 associations seen here reflect early anatomic substrates of AD; this sample does not currently have AD-relevant data to address this possibility.

A significant effect was found for *g*_*fa*_. This suggests that the *TOMM40* 523 repeat may influence processes that are sufficiently pleiotropic to affect general white matter integrity. Lopez et al. (2012) conducted a pathway analysis on *g*_*fa*_ based on the current sample dataset (n = 535). The authors used the web-based gene set enrichment analysis toolkit (WebGestalt; Duncan et al., 2010; Zhang et al., 2005) to conduct enrichment analysis for gene ontology on genes that were significantly associated with *g*_*fa*_ (n = 173 genes, *p* < 0.01). Twenty-three ontology categories had enriched gene numbers, the most significant of which were “calcium-dependent cell-cell adhesion” (*p* = 1.15 × 10^−11^) and “synapse assembly” (*p* = 5.20 × 10^−8^). Speculatively, the nominally significant association between *TOMM40* 523 repeat length and *g*_*fa*_ may relate to these ontologies. Lopez et al. (2012) found that *TOMM40* did not reach gene significance based on 19 SNPs; note, however, that report did not directly assess variation in the poly-T repeat locus.

Significant effects of *TOMM40* 523 length were found only in the *APOE* ε3/ε4 genotype. The S allele may modulate the toxic effects of *APOE* ε4 allele presence (Bruno et al., 2011). Crenshaw et al. (2012) report a protective effect of the S allele on the age of onset of mild cognitive impairment in ε3/ε4 participants, but in contrast a deleterious effect of the S/S genotype > S/VL genotype > VL/VL genotype in the ε3/ε3 and ε2/ε3 genotype. The effect was significant on age of onset distributions in 106 conversion events using standard neuropsychological tests in a series of 508 Caucasians ascertained prospectively. It is unclear what drives these differing directions; opposing deleterious, protective, and/or null effects of the S allele have been reported in different studies of AD risk, brain structure, and/or cognitive aging phenotype reports (e.g., Cruchaga et al., 2011; Johnson et al., 2011; Schiepers et al., 2012). Further study of poly-T repeat biological function and significance is required in large samples, including those with AD, *APOE*, or age of disease onset data/or age of disease onset data (Bekris et al., 2012; Cruchaga et al., 2011; Hedskog et al., 2012).

### 5.4 Summary

The present study found relatively circumscribed nominal significant effects of the *APOE* ε and *TOMM40* 523 gene loci on brain white matter integrity in the LBC1936. Most of these associations attenuated when corrected for multiple testing. While this study is relatively large for a brain imaging and/or genetics report, replication in large independent samples is required.

## Disclosure statement

Allen. D. Roses is the CEO and only stock-holder of Zinfandel Pharmaceuticals, a company in an Alliance with Takeda Pharmaceuticals, to perform the prospective qualification of the *TOMM40* marker for age of onset distribution of Alzheimer's disease. For this study, Zinfandel Pharmaceuticals paid for the *TOMM40* assays to be performed for medical research, not as a clinical diagnostic. Ann. M. Saunders is the spouse of Allen. D. Roses, and Ann. M. Saunders and Michael. W. Lutz are consultants to Zinfandel Pharmaceuticals.

## Figures and Tables

**Table 1:** Apolipoprotein-ε (*APOE*) and white matter integrity

White matter tract (FA)	Step 1 ε4 allele presence (vs. absence)			Step 2 *APOE* ε2/ε3 and ε2/ε2 (vs. ε3/ε3)		
	(df) F statistics	*p*	Partial η^2^	(df) F statistics	*p*	Partial η^2^
General factor (*g*_*fa*_)	(1, 524) = 0.83	0.362	0.002	(1, 376) = 0.12	0.730	0.000
Genu of the corpus callosum	(1, 559) = 0.20	0.659	0.000	(1, 399) = 0.26	0.612	0.001
Splenium of the corpus callosum	(1, 575) = 0.07	0.788	0.000	(1, 409) = 0.00	0.985	0.000
Left arcuate fasciculus	(1, 552) = 0.00	0.987	0.000	(1, 393) = 0.62	0.432	0.002
Right arcuate fasciculus.	(1, 500) = 0.17	0.683	0.000	(1, 356) = 0.03	0.873	0.000
Left anterior thalamic radiation	(1, 479) = 0.00	0.962	0.000	(1, 341) = 0.00	0.957	0.000
Right anterior thalamic radiation	(1, 556) = 0.11	0.745	0.000	(1, 395) = 0.00	0.979	0.000
Left uncinate fasciculus.	(1, 492) = 1.57	0.212	0.003	(1, 348) = 0.00	0.956	0.000
Right uncinate fasciculus.	(1, 545) = 0.00	0.990	0.000	(1, 390) = 0.03	0.873	0.000
Left rostral cingulum	(1, 556) = 0.06	0.803	0.000	(1, 394) = 0.27	0.604	0.001
Right rostral cingulum	(1, 564) = 0.09	0.760	0.000	(1, 402) = 0.28	0.603	0.001
Left ventral cingulum	(1, 561) = 2.36	0.125	0.004	(1, 396) = 1.05	0.305	0.003
Right ventral cingulum	*(1, 570)* = *5.48*	*0.020*	*0.010*	(1, 404) = 1.19	0.277	0.003
Left inferior longitudinal fasciculus	*(1, 575)* = *5.04*	*0.025*	*0.009*	(1, 407) = 0.17	0.689	0.000
Right inferior longitudinal fasciculus	(1, 577) = 0.27	0.604	0.000	(1, 409) = 1.07	0.303	0.003

Age in days at the time of testing and gender is statistically controlled. Associations significant at *p* < 0.05 are given italics. Key: df, degrees of freedom; FA, fractional anisotropy.

**Table 2:** Translocase of outer membrane 40 (*TOMM40*) “523” length and white matter integrity

White matter tract (FA)	Whole sample			*APOE* ε3/ε4 genotype			*APOE* ε3/ε3 genotype		
	(df) F statistics	*p*	Partial η^2^	(df) F statistics	*p*	Partial η^2^	(df) F statistics	*p*	Partial η^2^
General factor (*g*_*fa*_)	(5, 529) = 2.11	0.063	0.020	*(1, 122)* = *4.95*	*0.028*	*0.039*	(2, 303) = 1.04	0.356	0.007
Genu of the corpus callosum	(5, 564) = 1.83	0.105	0.016	(1, 131) = 2.42	0.122	0.018	(2, 321) = 0.92	0.401	0.006
Splenium of the corpus callosum	(5, 580) = 0.20	0.963	0.002	(1, 136) = 1.56	0.214	0.011	(2, 331) = 0.21	0.810	0.001
Left arcuate fasciculus	(5, 557) = 0.64	0.669	0.006	(1, 131) = 0.92	0.339	0.007	(2, 317) = 0.62	0.539	0.004
Right arcuate fasciculus.	(5, 507) = 0.50	0.774	0.005	(1, 117) = 0.30	0.584	0.003	(2, 289) = 0.84	0.435	0.006
Left anterior thalamic radiation	(5, 481) = 1.68	0.139	0.017	(1, 110) = 2.68	0.104	0.024	(2, 277) = 0.19	0.826	0.001
Right anterior thalamic radiation	(5, 560) = 1.47	0.197	0.013	(1, 132) = 3.22	0.075	0.024	(2, 320) = 2.06	0.129	0.013
Left uncinate fasciculus.	(5, 594) = 1.61	0.157	0.016	*(1, 117)* = *4.16*	*0.044*	*0.034*	(2, 279) = 0.49	0.616	0.003
Right uncinate fasciculus.	(5, 549) = 1.13	0.343	0.010	(1, 128) = 1.39	0.240	0.011	(2, 314) = 0.88	0.416	0.006
Left rostral cingulum	(5, 561) = 2.09	0.065	0.018	*(1, 133)* = *5.51*	*0.020*	*0.040*	(2, 317) = 1.87	0.156	0.012
Right rostral cingulum	*(5, 568)* = *2.99*	*0.011*	*0.026*	(1, 133) = 1.96	0.164	0.015	(2, 326) = 2.87	0.058	0.017
Left ventral cingulum	*(5, 564)* = *2.56*	*0.025*	*0.022*	*(1, 136)* = *13.35*	*<0.001*	*0.089*	(2, 322) = 0.72	0.487	0.004
Right ventral cingulum	(5, 576) = 0.75	0.584	0.006	(1, 137) = 0.00	0.980	0.000	(2, 326) = 0.83	0.439	0.005
Left inferior longitudinal fasciculus	(5, 580) = 0.88	0.492	0.008	(1, 138) = 0.12	0.725	0.001	(2, 330) = 0.83	0.436	0.995
Right inferior longitudinal fasciculus	(5, 581) = 0.66	0.655	0.006	(1, 138) = 0.37	0.547	0.003	(2, 331) = 0.81	0.448	0.005

Age in days at the time of testing and gender is statistically controlled. Associations significant at *p* <0.05 are given italics. Key: df, degrees of freedom; FA, fractional anisotropy.

**Table 3:** Apolipoprotein-e (*APOE*), translocase of outer membrane 40 (*TOMM40*) ‘523’ loci, and white matter integrity; nominally significant findings (*p* < 0.05 in Table 1, and Table 2 subgroup analyses) adjusted for additional covariates

	White matter tract (FA)	Adjusted for age 11 IQ							Additionally adjusted for vascular disease history						
		(df) F statistics	*p*	Partial η^2^	Contrasts				(df) F statistics	*p*	Partial η^2^	Contrasts			
					n	Est. Mean (95% CI)	n	Est. Mean (95% CI)				n	Est. Mean (95% CI)	n	Est. Mean (95% CI)
*APOE*					_______ε4+______		_______ε4-_______					_______ε4+______		_______ε4-_______	
ε4 + versus ε4 − comparison	Right ventral cingulum	(1, 535) = 4.17	0.042	0.008	156	0.285 (0.279; 0.192)	384	0.293 (0.289; 0.297)	(1, 530) = 3.90	0.049	0.007	156	0.285 (0.279; 0.292)	384	0.293 (0.289; 0.297)
	Left inferior longitudinal fasciculus	(1, 539) = 7.30	0.007	0.013	157	0.392 (0.385; 0.399)	387	0.404 (0.399; 0.408)	(1, 534) = 7.00	0.008	0.013	157	0.392 (0.385; 0.399)	387	0.404 (0.399; 0.408)
*TOMM40*					_______S/L*_______		_______L*/L*_______					_______S/L*_______		_______L*/L*_______	
‘523’ poly-T repeat length genotype, in *APOE* ε3/ε4 subgroup	General factor (*g*_*fa*_)	(1, 114) = 5.35	0.023	0.045	67	−0.295 (−0.543; −0.047)	52	0.143 (−0.138; 0.425)	(1, 109) = 5.84	0.017	0.051	67	−0.309 (−0.559; −0.059)	52	−0.161 (−0.124; 0.446)
	Left uncinate fasciculus	(1, 108) = 3.68	0.058	0.033	65	0.325 (0.317; 0.333)	48	0.337 (0.328; 0.346)	(1, 112) = 4.12*	0.045*	0.035*	68	0.324 (0.317; 0.332)	53	0.337 (0.328; 0.345)
	Left rostral cingulum	(1, 124) = 5.50	0.021	0.042	71	0.428 (0.417; 0.439)	58	0.447 (0.435; 0.459)	(1, 119) = 5.83	0.017	0.047	71	0.428 (0.417; 0.439)	58	0.448 (0.436; 0.460)
	Left ventral cingulum	(1, 125) = 14.40	<0.001	0.103	71	0.280 (0.271; 0.289)	59	0.306 (0.296; 0.315)	(1, 120) = 15.75	<0.001	0.116	71	0.279 (0.270; 0.316)	59	0.307 (0.297; 0.316)

Age in days at the time of testing and gender is statistically controlled. *Age-11 IQ not included as a covariate. Key: CI, confidence interval; df, degrees of freedom; Est. mean, estimated marginal mean difference adjusted for applicable covariates; FA, fractional anisotropy; “L*”, pooled “long” and “very-long” alleles; *TOMM40* 523 “S”, “short” allele.
