# Supplementary material for: Alzheimer's disease susceptibility genes APOE and TOMM40, and brain white matter integrity in the Lothian Birth Cohort 1936
Source: Neurobiol Aging. 2014 Jun;35(6):1513.e25–33. doi: 10.1016/j.neurobiolaging.2014.01.006 (PMC3969262; doi:10.1016/j.neurobiolaging.2014.01.006)
Supplement: Supplementary Table 1 [file mmc1.docx]

**Supplementary Table 1** Demographic data for the current sample, split by Apolipoprotein-e (*APOE*) and translocase of outer membrane 40 *(TOMM40)* ‘523’ genotypes.

|  | *APOE* genotype | | | | | | |
| --- | --- | --- | --- | --- | --- | --- | --- |
|  | ε2/ε2 | ε2/ε3 | ε2/ε4 | ε3/ε3 | ε3/ε4 | | ε4/ε4 |
| N | 2 | 77 | 14 | 376 | 160 | | 13 |
| Gender (males/females) | 2/0 | 41/36 | 7/7 | 194/182 | 86/74 | | 7/6 |
| Age in years (SD) | 73.82  (0.34) | 72.85  (0.68) | 72.76  (0.93) | 72.66  (0.70) | 72.63  (0.74) | | 72.49  (0.90) |
| Age 11 IQ (SD) | 82.60  (24.45) | 100.83  (14.85) | 98.93  (17.57) | 101.46  (13.83) | 101.79  (14.24) | | 104.86  (16.76) |
| MMSE score (SD) | 27.00 (4.24) | 28.68  (1.44) | 28.29  (1.73) | 28.83  (1.26) | 28.90  (1.20) | | 28.69  (1.49) |
|  | *TOMM40* ‘523’ poly-T repeat genotype | | | | | | |
|  | S/S | S/L | S/VL | L/L | L/VL | VL/VL | |
| N | 102 | 94 | 240 | 15 | 76 | 125 | |
| Gender (males/females) | 57/45 | 51/43 | 118/122 | 7/8 | 38/38 | 66/59 | |
| Age in years (SD) | 72.88  (0.72) | 72.59  (0.76) | 72.75  (0.72) | 72.31  (0.90) | 72.71  (0.78) | 72.60  (0.67) | |
| Age 11 IQ (SD) | 98.84  (15.17) | 102.72  (13.31) | 101.44  (13.96) | 105.96  (15.93) | 101.01  (15.46) | 103.19  (13.29) | |
| MMSE score (SD) | 28.71  (1.44) | 28.76  (1.30) | 28.80  (1.20) | 28.80  (1.47) | 28.88  (1.22) | 28.98  (1.25) | |
|  | *TOMM40* ‘523’ poly-T repeat genotype  (in  *APOE ε3/ε3* subgroup) | | | | | | |
|  | S/S |  | S/L* |  | L*/L* |  | |
| N | 76 |  | 199 |  | 95 |  | |
| Gender (males/females) | 43/33 |  | 94/105 |  | 54/41 |  | |
| Age in years (SD) | 72.62  (0.71) |  | 72.73  (0.72) |  | 72.52  (0.66) |  | |
| Age 11 IQ (SD) | 100.07  (15.13) |  | 102.15  (13.62) |  | 101.49  (13.18) |  | |
| MMSE score (SD) | 28.82  (1.40) |  | 28.86  (1.13) |  | 28.87  (1.29) |  | |
|  | *TOMM40* ‘523’ poly-T repeat genotype  (in  *APOE ε3/ε4* subgroup) | | | | | | |
|  | S/L* | |  | L*/L* | |  | |
| N | 86 | |  | 72 | |  | |
| Gender (males/females) | 47/39 | |  | 37/35 | |  | |
| Age in years (SD) | 72.60  (0.71) | |  | 72.66  (0.79) | |  | |
| Age 11 IQ (SD) | 101.49  (13.79) | |  | 102.38  (14.90) | |  | |
| MMSE score (SD) | 28.80  (1.25) | |  | 28.99  (1.14) | |  | |
| *Note.* SD = standard deviation, *TOMM40* 523 ‘S’ = ‘Short’ allele, ‘L*’ = pooled ‘Long’ and ‘Very-long’ alleles. | | | | | | | |
